# Efficient Nonviral Gene Therapy Using Folate-Targeted Chitosan-DNA Nanoparticles *In Vitro*

**DOI:** 10.5402/2012/369270

**Published:** 2012-03-07

**Authors:** Christian Jreyssaty, Qin Shi, Huijie Wang, Xingping Qiu, Françoise M. Winnik, Xiaoling Zhang, Kerong Dai, Mohamed Benderdour, Julio C. Fernandes

**Affiliations:** ^1^Orthopaedics Research Laboratory, Research Center, Sacré-Coeur Hospital, University of Montreal, 5400 West Gouin Boulevard, Montreal, QC, Canada H4J 1C5; ^2^Faculty of Pharmacy and Department of Physical Chemistry and Polymer Science, University of Montreal, Pavillon J.A. Bombardier, C.P. 6128, Succursale Centre-ville, Montreal, QC, Canada H3C 3J7; ^3^Orthopaedic Cellular and Molecular Biology Laboratory, Institute of Health Sciences, Chinese Academy of Sciences and Shanghai Jiao Tong University School of Medicine, 225 South Chongqing Road, Shanghai 200025, China

## Abstract

Nonviral cationic polymers like chitosan can be combined with DNA to protect it from degradation. The chitosan is a biocompatible, biodegradable, nontoxic, and cheap polycationic polymer with low immunogenicity. The objective of this study was to synthesize and then assess different chitosan-DNA nanoparticles and to select the best ones for selective *in vitro* transfection in human epidermoid carcinoma (KB) cell lines. It revealed that different combinations of molecular weight, the presence or absence of folic acid ligand, and different plasmid DNA sizes can lead to nanoparticles with various diameters and diverse transfection efficiencies. The intracellular trafficking, nuclear uptake, and localization are also studied by confocal microscopy, which confirmed that DNA was delivered to cell nuclei to be expressed.

## 1. Introduction

Gene therapy is being applied to various health problems, such as cancer, acquired immunodeficiency syndrome, and cardiovascular diseases. The main challenge is to develop a method that delivers the transgene to selected cells, where a proper gene expression can be achieved. Several trials have aimed at introducing genes straight into human cells, focusing on diseases caused by single-gene defects, such as cystic fibrosis [1], hemophilia [2], adenosine deaminase deficiency [3], muscular dystrophy [4], and sickle cell anemia [5]. 

Ideally, gene therapy must protect DNA against degradation by nucleases in intercellular matrices so that the disposition of macromolecules is not affected. Transgenes should be brought across the plasma membrane and into the nucleus of targeted cells but should have no detrimental effects. Hence, interaction with blood components, vascular endothelial cells, and uptake by the reticuloendothelial system must be avoided [6]. 

For gene therapy to succeed, small-sized systems must internalize into cells and pass to the nucleus. Also, flexible tropisms allow applicability to a range of disease targets. Last but not least, such systems should be able to escape endosome-lysosome processing for endocytosis [7]. 

Viral gene therapy consists of using viral vectors which, given their structure and mechanisms of action, are good candidates or models to carry therapeutic genes efficiently, leading to long-term expression [8, 9]. They have the natural ability to enter cells and express their own proteins. Nowadays, most viral vectors used are retroviruses, herpes virus, adenoviruses, and lentiviruses [10]. However, viral vectors can cause several problems to patients, namely, toxicity, oncogenic effects, and immune and inflammatory responses. Because of safety and cost concerns, nonviral vectors have gained a lot of attention in the literature [6, 7].

Nonviral gene therapy has been explored by physical approaches (transfer by gene gun, electroporation, ultrasound-facilitated and hydrodynamic delivery) as well as chemical approaches (cationic lipid-mediated gene delivery, and cationic polymer-mediated gene transfer) [11].

Synthetic and natural cationic polymers (positively charged) have been widely used to carry DNA (negatively charged) and condense it into small particles, facilitating cellular internalization via endocytosis through charge-charge interactions with anionic sites on cell surfaces. In this category, we include polyethylenimine, polyamidoamine, and polypropylamine dendrimers, polyallylamine, chitosan, cationic dextran, cationic peptides, and proteins [6, 12]. Nonviral delivery systems for gene therapy have been increasingly proposed as safer alternatives to viral vectors because they evoke a minimal host immune response, are stable in storage, easy to produce in large quantities, and are of low cost [13]. 

Chitosan (Ch) is a biocompatible, biodegradable, nontoxic, and cheap polycationic polymer with low immunogenicity. Positively charged Ch can be easily complexed with DNA and protected from nuclease degradation [7, 14]. It is also hypoallergenic and has natural antibacterial properties. Sonication and organic solvents are not required for its preparation, which minimizes possible damage to DNA during complexation. Ch (*α* (1→4) 2-amino-2-deoxy-*β*-D-glucan) is produced commercially by deacetylation of chitin, the structural element in the exoskeleton of crustaceans (e.g., crab, shrimp) [15, 16]. This polymer has a weak base; consequently, in acidic media, the amine groups will be positively charged, conferring high-charge density to the polysaccharide [7]. The cationic characteristic of chitosan is a crucial parameter for complex formation. 

Folic acid (FA) receptors (FR) are overexpressed on many human cancer cell surfaces, and the nonepithelial isoform FR(*β*) is expressed on activated synovial macrophages present in large numbers in rheumatoid arthritis (RA) [17].

FA-mediated transfection has been shown to facilitate DNA internalization into cells through membrane receptors both *in vitro *and *in vivo*. Another strategy for improving transfection is to take advantage of the mechanism of folate uptake by cells to promote targeting and internalization, hence improving transfection efficiency [18]. FA is appealing as a ligand for targeting cell membranes and allowing nanoparticle endocytosis via FR for higher transfection yields. Importantly, the high affinity of folate for binding to its receptor (1 nm) and its small size make it ideal for specific cell targeting. Moreover, the ability of FA to bind its receptor for endocytosis is not altered by covalent conjugation of small molecules [19].

The objective of this study was to synthesize and then assess different Ch nanoparticles and to select the best suited ones for selective *in vitro* transfection in human epidermoid carcinoma (KB) cell lines. 

## 2. Materials and Methods

### 2.1. Synthesis of Ch Nanoparticles

Low-molecular-weight (LMW) Ch (Wako-10, degree of deacetylation (DDA) = 89%, amine phosphate ratio N : P of 3 : 1) was purchased from Wako Chemicals (Richmond, VA, USA). Ch of different molecular weights (MWs) (5 kDa: Ch5, 25 kDa: Ch25, and 50 kDa: Ch50) were selected to create different nanoparticles.

FA (MW = 441.41 Da) was purchased from Sigma-Aldrich Chemicals (St. Louis, MO, USA). *α*-amino, *ω*-carboxyl poly(ethylene glycol) (NH_2_-PEG-COOH, MW = 3,400 Dalton) was obtained from Shearwater Inc. (Dallas, Texas, USA). Ch was further deacetylated by treatment with concentrated NaOH solution (50%) to obtain ~89% deacetylation according to a reported procedure [20]. 

To prepare Ch-PEG-folate conjugate, FA was combined with Ch through the PEG arm. FA was first attached to NH_2_-PEG-COOH via well-known carbodiimide chemistry to give folate-PEG-COOH. Then, folate-PEG-COOH was again activated by *N*,*N *′-dicyclohexylcarbodiimide and *N*-hydroxysuccinimide (NHS) for conversion to the reactive intermediate folate-PEG-CO-NHS and subsequently grafted onto Ch to achieve Ch-PEG-folate conjugate [21]. The level of folate-PEG incorporation was determined to be 1.1 mol% with respect to the glucosamine unit of chitosan by UV-Vis spectroscopy using FA as standard. (The extinction coefficient (*λ*_363nm_) of FA was 6,165 M^−1 ^cm^−1^ in pH 7.4 phosphate buffer (0.1 M).) A stock solution of 0.1% Ch or Ch-PEG-FA was prepared in 25 mM acetic acid, under continuous stirring at 37°C, and then adjusted to pH 5.0 with 1 M NaOH. 100 *μ*g/mL DNA solution was buffered in 43 mM Na_2_SO_4_ [22]. 

The plasmids used in this study were VR1412 and pEGFP-C3. pEGFP-C3 is a nonviral, mammalian plasmid. It has a CMV promoter and backbone size of 4,727 bp. It codes for GFP (green fluorescent protein) that allows researchers to optically detect specific types of cells *in vitro* or *in vivo* [23].

The plasmid DNA VR1412, encoding the *β*-galactosidase (*β*-gal) reporter gene with a CMV promoter and a backbone size of 8,100 kb, was obtained from VICAL Inc. (San Diego, CA, USA) [23, 24]. 

Mass quantity plasmids (VR1412 and pEGFP-C3) were prepared with commercial kits (Mega Kits, Qiagen Mississauga, ON, Canada), and finally suspended in sterile water. The integrity of DNA plasmid was analyzed by 0.8% agarose gel electrophoresis and quantified (DNA concentration) by UV spectrometry at 260 nm. All procedures were undertaken according to manual instructions [25]. 

Ch solutions were heated at 55°C for 1 minute; then, nanoparticle synthesis was carried out by mixing an equal volume of Ch and DNA stock solutions at room temperature (Ch : DNA ratio = 1 : 1), stirred for 30 minutes and allowed to stand for 1 hour before transfection [25]. Four types of nanoparticles were synthesized: Ch-GFP, Ch-PEG-FA-GFP, Ch-*β*-gal, and Ch-PEG-FA-*β*-gal. Ch5, Ch25, and Ch50 are used for each type, giving a total of 12 different nanoparticle complexes. Ch nanoparticles were compared to Lipofectamine 2000 (LF), a commercially available lipid vector, which is considered to be the gold standard for its high transfection efficiency. LF was combined with DNA by mixing with a respective volume ratio of 1 : 3.25 *μ*L at room temperature. All measurements were collected in triplicate, and each experiment repeated 3 times [7]. 

### 2.2. Electrophoresis Gel Analysis

Agarose gel electrophoresis is a technique that separates DNA or RNA molecules by size. It is performed by moving negatively charged nucleic acid molecules through an agarose matrix with an electric field. 

Electrophoresis gel analysis contributes to the assessment of gene condensation with Ch and DNA integrity. Intact and complexed genes are seen and analyzed [25].

### 2.3. Nanoparticle Size

We measured the nanoparticle sizes of Ch-GFP, Ch-PEG-FA-GFP, Ch-*β*-gal, and Ch-PEG-FA-*β*-gal with Ch5, Ch25, and Ch50 kDa in an ALV/CGS-3 Compact Goniometer System for dynamic light scattering fixed at a 90° angle with a wavelength of *λ* = 632.8 nm. The results indicated the distribution curve and diameter of the nanoparticles in nm [16].

### 2.4. Zeta Potential

Zeta potential allows the measurement of overall surface charge of nanoparticles, which represents a critical factor in their interaction with cellular membranes. This study was performed at 25°C, using a Malvern Zetasizer 4 (Malvern Instruments Ltd., Malvern, UK) and green disposable cuvettes for the zetasizer and nanoseries. 

### 2.5. Cell Preparation for *In Vitro* Transfection

In this study, we selected KB cells for their unlimited division capacity and overexpression of FR [26]. The cells were obtained from the American Type Culture Collection (Rockville, MD, USA). They were inoculated at a density of 14 × 10^5^ cells/well (in 6-well plates) with RPMI medium 1640 containing 10% of fetal bovine serum (FBS) and 1% penicillin. The plates were kept in a cell culture incubator under 5% CO_2_ at 37°C. 

### 2.6. Cell Transfection

*In vitro* transfection efficiency was undertaken in KB cells incubated in 6-well plates with 600 *μ*L of Ch nanoparticle complexes (Ch-GFP, Ch-PEG-FA-GFP, Ch-*β*-gal, and Ch-PEG-FA-*β*-gal) with Ch5, Ch25 and Ch50 kDa. The cells are incubated for 2 hours at 37°C with 1,400 *μ*L of fresh antibiotic-free RPMI medium 1640, serum, and FA, then examined by optical microscopy. 

Positive (cells transfected with LF or DNA) and negative (nontransfected cells) controls were used. Gene expression was detected 48 hours after transfection [7].

### 2.7. GFP Expression

GFP is composed of 238 amino acids (26.9 kDa), and fluoresces green when exposed to blue light under a fluorescence microscope equipped with an analog camera. In cellular and molecular biology, it is frequently deployed as a reporter of gene expression. 

The fluorescence emitted by KB cells showing GFP expression was detected with Photon Technology International at an excitation wavelength of 484 nm and emission of 510 nm.

Each sample was measured 10 times. Numerical results were analyzed by FeliX 1.42.exe. Photos of cells were taken to complete and confirm the quantitative results [13]. 

### 2.8. *β*-Gal Expression

The VR1412 gene codes for *β*-gal, an enzyme that catalyzes the hydrolysis of *β*-galactosides into monosaccharides. 

*β*-gal activity was measured with Sigma kits (Sigma-Aldrich, Oakville, ON, Canada) and compared to total protein (pg *β*-gal per mg of cellular protein).

Total protein content in the solution was quantified by bicinchoninic acid assay (Pierce, Rockford, IL, USA). In this assay, 2 molecules of bicinchoninic acid chelated a single Cu1+ ion, forming a purple water-soluble complex that strongly absorbed light at 562 nm.

The results of *β*-gal expression were read on 96-well plates by a EL-800 Universal Microplate Reader (Bio-Tek Instruments Inc., Winooski, VT, USA) at a wavelength of 420 nm [25]. 

### 2.9. Cell Viability

Cytotoxicity (nanoparticle effects on cell viability) was studied by 3-[4,5-dimethylthiazol-2-yl]-2,5diphenyl tetrazolium bromide assay, a standard colorimetric assay for measuring the activity of enzymes that reduce yellow MTT to formazan in living cell mitochondria, giving it a purple color. The absorbance of this colored solution can be quantified by measurement with a spectrophotometer at a certain wavelength (usually between 500 and 600 nm). Coloration intensity was proportional to the number of living cells because reduction takes place only when mitochondrial reductase enzymes are active. 

Viability was measured with the EL-800 Universal Microplate Reader at 570 nm, in 96-well plates [25].

### 2.10. Intracellular Trafficking, Nuclear Uptake, and Localization

KB cells were incubated with Ch25-PEG-FA-*β*-gal nanoparticle complexes for 2 hours at 37°C, fixed, and examined by confocal microscopy. Plasmid DNA was bound with propidium iodide (PI) (red), and stained DNA was purified before nanoparticle synthesis. Endosomes and lysosomes were immunolabeled with antiearly endosome maker, lysosomal-associated membrane protein-1, and fluorescent-labeled anti-mouse antibodies (green). DAPI staining located cell nuclei (blue). 

### 2.11. Statistical Analyses

All values were expressed as means ± standard deviation and analyzed by unpaired Student's *t-*test and/or one-way ANOVA with *P* ≤ 0.05 considered as a statistically significant difference. 

## 3. Results

### 3.1. Synthesis of Folate-PEG-Chitosan

**Figure 1:**
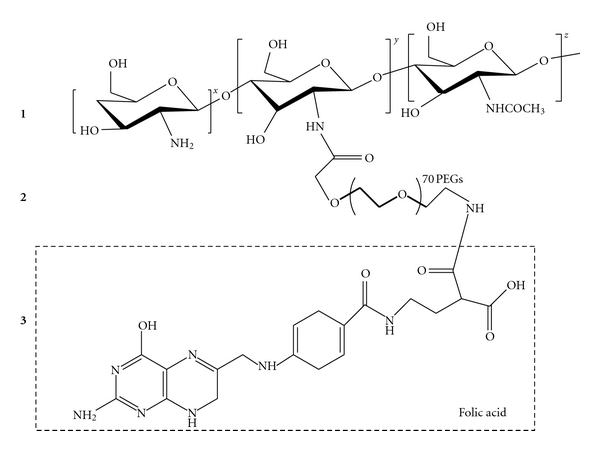
Molecular grafted structure of chitosan-PEG-FA complex. (1) Chitosan; (2) PEG; (3) folic acid.

The feasibility of folate-mediated targeting of chitosan was investigated after FA coupling to chitosan with PEG as spacer. The structure of Folate-PEG-chitosan conjugate is illustrated in Figure 1. 

### 3.2. Nanoparticle Characterization

**Figure 2:**
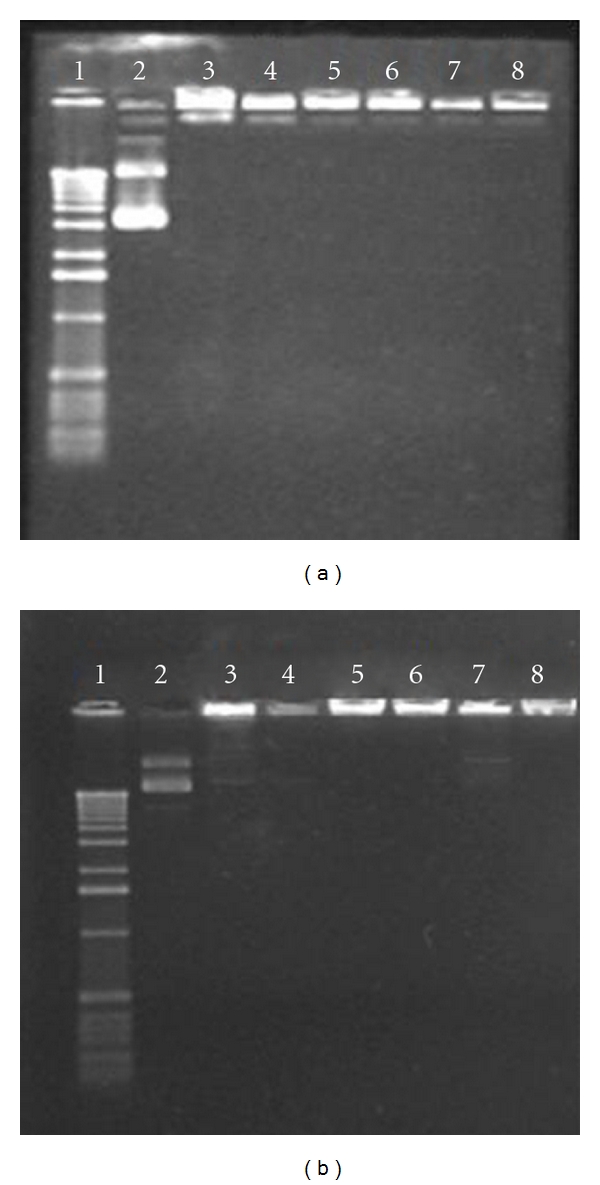
Agarose gel electrophoresis. (a) Chitosan nanoparticles with VR1412 *β*-gal plasmid DNA. (b) Chitosan nanoparticles with GFP plasmid DNA. Lane 1: ladder; lane 2: naked DNA; lane 3: Ch-DNA nanoparticule with a Mw = 5 KDa chitosan; lane 4: Ch-PEG-FA-DNA nanoparticule with a Mw = 5 KDa chitosan; lane 5: Ch-DNA nanoparticule with a Mw = 25 KDa chitosan; lane 6: Ch-PEG-FA-DNA with a Mw = 25 KDa chitosan; lane 7: Ch-DNA with a Mw = 50 KDa chitosan; lane 8: Ch-PEG-FA-DNA nanoparticule with a Mw = 50 KDa chitosan.

**Figure 3:**
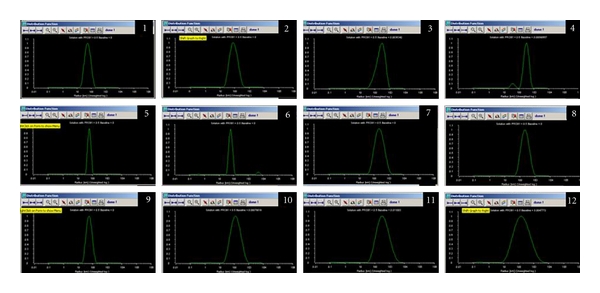
Distribution function of nanoparticles sizes (diameter in nm). (1) Ch5-*β*-gal: 114.27 ± 20.08 nm; (2) Ch5-PEG-FA-*β*-gal: 150.03 ± 9.76 nm; (3) Ch5-GFP: 223.83 ± 11.54 nm; (4) Ch5-PEG-FA-GFP: 278 ± 27.22 nm; (5) Ch25-*β*-gal: 127.67 ± 16.21 nm; (6) Ch25-PEG-FA-*β*-gal: 134.84 ± 14.13 nm; (7) Ch25-GFP: 151.43 ± 9.35 nm; (8) Ch25-PEG-FA-GFP: 204.33 ± 5.91 nm; (9) Ch50-*β*-gal: 111.94 ± 20.75 nm; (10) Ch50-PEG-FA-*β*-gal: 247.34 ± 18.33 nm; (11) Ch50-GFP: 160.21 ± 6.82 nm; (12) Ch50-PEG-FA-GFP: 302.08 ± 34.13 nm (*P* < 0.05, *n* = 5).

**Figure 4:**
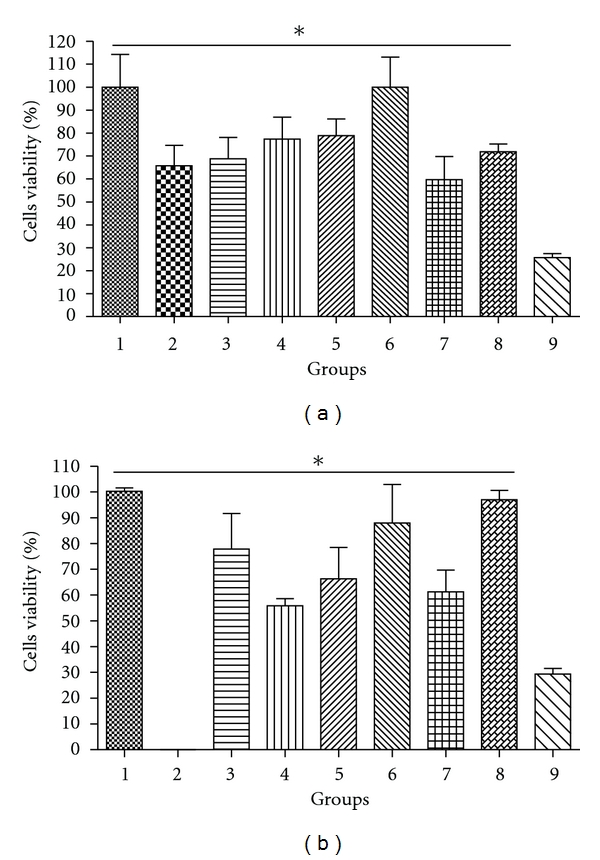
Cells viability treated with chitosan nanoparticules (a) containing the VR1412 *β*-gal plasmid DNA. Group 1: negative control (KB cells); Group 2: naked DNA; Group 3: Ch-DNA nanoparticule with a Mw = 5 KDa chitosan; Group 4: Ch-PEG-FA-DNA nanoparticule with a Mw = 5 KDa chitosan; Group 5: ch-DNA nanoparticule with a Mw = 25 KDa chitosan; Group 6: Ch-PEG-FA-DNA with a Mw = 25 KDa chitosan; Group 7: Ch-DNA with a Mw = 50 KDa chitosan; Group 8: Ch-PEG-FA-DNA nanoparticule with a Mw = 50 KDa chitosan; Group 9: Lipofectamine. (b) Chitosan nonconjugated with DNA, Group 1: negative control; Group 3: Mw = 5 KDa chitosan; Group 4: Mw = 5 KDa chitosan combined with FA; Group 5: Mw = 25 KDa Chitosan; Group 6: Mw = 25 KDa chitosan combined with FA; Group 7: Mw = 50 KDa chitosan; Group 8: Mw = 50 KDa chitosan combined with FA; Group 9: lipofectamine. *Statistical significant differences compared with positive control (*P* < 0.05).

**Figure 5:**
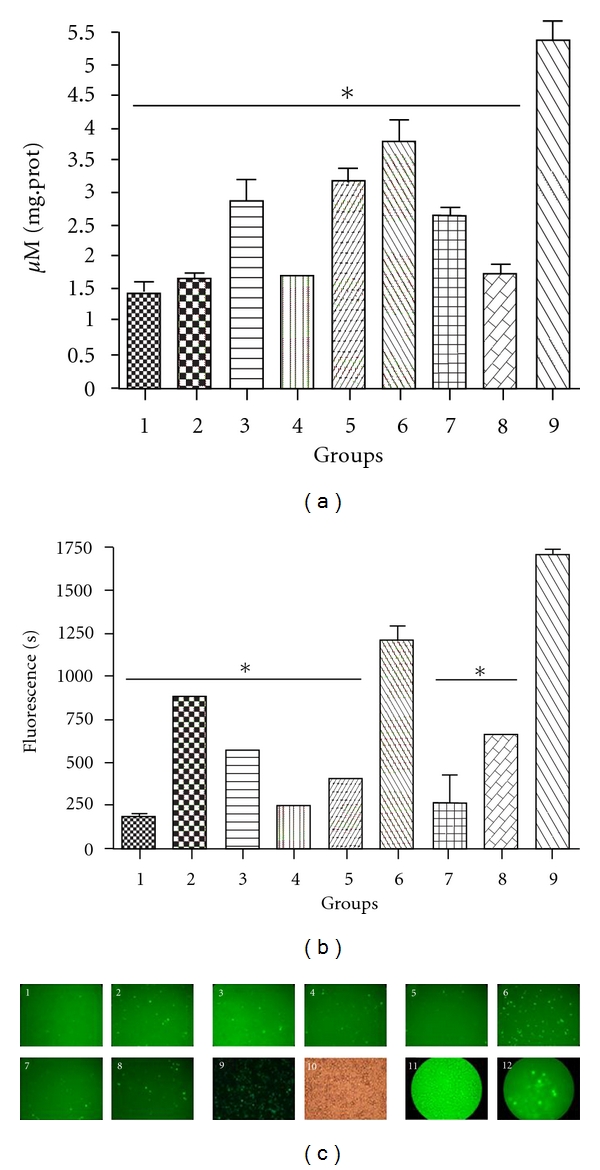
Expression of (a) VR1412 *β*-gal, (b) GFP in KB cells and (c) KB cells expressing GFP seen with fluorescent microscopy (magnification × 10) Group 1: negative control (KB cells); Group 2: Naked DNA; Group 3: Ch-DNA nanoparticule with a Mw = 5 KDa chitosan; Group 4: Ch-PEG-FA-DNA nanoparticule with a Mw = 5 KDa chitosan; Group 5: Ch-DNA nanoparticule with a Mw = 25 KDa chitosan; Group 6**: **Ch-PEG-FA-DNA with a Mw = 25 KDa chitosan; Group 7: Ch-DNA with a Mw = 50 KDa chitosan; Group 8: Ch-PEG-FA-DNA nanoparticulewith a Mw = 50 KDa chitosan; Group 9: lipofectamine coupled with DNA; Group 10: KB cells seen with optical microscopy; Group 11: KB cells seen with fluorescent microscopy; Group 12: negative control, nontreated KB cells seen with fluorescent microscopy. *Statistical significant differences compared with positive control (*P* < 0.05).

**Figure 6:**
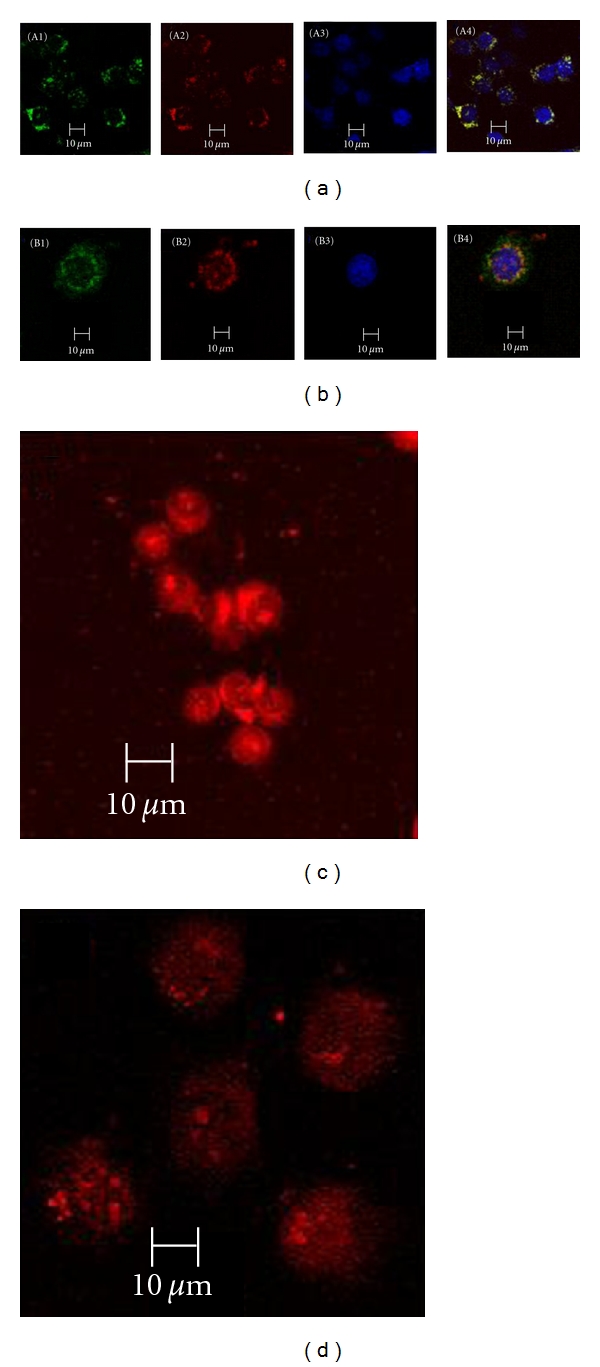
Intracellular trafficking in KB cells. KB cells were incubated with chitosan nanoparticle complex for 2 hours 37°C, fixes and examined by confocal microscopy. A1 and B1: endosomes and lysosomes immunolabeled with antiearly endosome maker (EEA-1) and lysosomal-associated membrane protein1 (LAMP-1) and fluorescent-labeled anti-mouse antibodies appear green. A2 and B2: plasmid DNA binded with Propidium Iodide (PI) appears red. Stained DNA were purified before nanoparticle; synthesis. A3 and B3: bleu located nuclei of cells as DAPI staining. Under the same condition of transfection with chitosan nanoparticule, A1, B1: endosomes and lysosomes A4 and B4. Superposition of different staining for a three-dimensional image reconstruction. (c) Whole cells, (d) nuclei of KB were isolated. Red shows plasmid DNA beforehand stained with PI; no any staining is there for nuclear DNA.

Agarose gel electrophoresis of *β*-gal (Figures 2(a)) and GFP (Figure 2(b)) as well as Ch-DNA and Ch-PEG-FA-DNA nanoparticles with different MW Ch presented dense bands, confirming complex formation (Figures 2(a) and 2(b)—3, 4, 5, 6, 7, 8). Particle sizes were found to be in the range of 100 to 300 nm, depending on the plasmid and presence/absence of FA (Figure 3). Zeta potential remained stable for all nanoparticles with average values around +15 mV. MTT viability studies revealed low cell toxicity compared to naked DNA and LF (Figure 4).

### 3.3. *In Vitro* Transfection Efficiency in KB Cells

Gene expression was most significant with Ch25-PEG-FA-DNA nanoparticles (Figures 5(a), 5(b), and 5(c)). *β*-gal expression was different among the various groups. Significant differences were observed when the experimental groups (3, 5, 6, and 7) were compared to the negative controls (1) (Figure 5(a)). 

By comparing the transfection rate using LF with the Ch25-PEG-FA-DNA nanoparticle, we found no significant difference (*P* = NS) between the 2 groups (Figure 5(b): column 6 and Figure 5(b): column 9).

Figure 5(c) illustrates the overall results and greater transfection efficiency of GFP gene expression in KB cells with Ch25-PEG-FA-DNA. The density of fluorescent cells compared to existing 14.10^5^ cells in each 6-well plate was an indicator of the transfection rate.

### 3.4. Intracellular Trafficking, Nuclear Uptake, and Localization

We localized endosomes and lysosomes stained in green, DNA in red, and nuclei in blue of KB cells transfected with Ch25-PEG-FA-*β*-gal nanoparticles. VR1412 plasmid bound PI and appeared in red. After nuclei isolation, we observed red, coloration, which confirmed that DNA was delivered to cell nuclei to be expressed (Figure 6).

## 4. Discussion

We have shown that biodegradable cationic polymers such as Ch have the potential for DNA complexation and may be used as nonviral vectors for gene therapy. The first aim of this study was to design and then evaluate Ch nanoparticles with different characteristics.

We also found Ch and Ch nanoparticles to be nontoxic in a range of toxicity tests. We also demonstrated the ability of Ch-DNA and Ch-PEG-FA-DNA complexes to condense and deliver plasmid DNA in human KB cells. 

In our study, transfection efficiency depended on Ch's MW, the presence or absence of FA, and the nature of the combined gene. Ch25-PEG-FA-DNA showed better GFP and *β*-gal expression *in vitro*. 

### 4.1. Nanoparticle Characteristics

Agarose gel electrophoresis confirmed the strong attachment of DNA to Ch and Ch-PEG-FA. The synthesis of Ch-DNA complexes was facilitated by attraction between free amino groups on the polymer and negatively charged phosphates found on DNA [24]. Lanes showing no unbound DNA explain the strong attachment of Ch-*β*-gal, Ch-GFP, Ch-PEG-FA-*β*-gal, and Ch-PEG-FA-GFP complexes. The interaction involved in the complexes was mainly electrostatic [7]. Previous studies suggested that covalent linkage of FA with Ch did not affect electrostatic attachment with DNA. After digestion with chitosanase and lysozyme, intact plasmid DNA was released from Ch [25], suggesting that the synthesis conditions and FA covalent linkage with Ch did not affect the integrity of condensed DNA. Usually, DNA has to maintain its supercoiled circular form for optimal gene expression. Nanoparticle complexes must provide DNA protection from physical, chemical, and enzymatic degradation. During the preparation of nanoparticles, there is a risk of damaging supercoiled DNA and converting it to linear or even fragmented DNA [27]. This was not observed in our experiments. 

We suggest that the size of 12 different nanoparticles depends on the size of the DNA molecule and the conjugate (PEG-FA) added to promote targeting and internalization on transfection. In the presence of FA attached to Ch through the PEG arm, we expected an increase in nanoparticle size. The MW of Ch did not significantly affect nanoparticle size. Also, zeta potential remained stable around +15 mV for all nanoparticle samples (N : P ratio of 3 : 1, DDA = 89%), and no impact on size was observed. 

The normal distribution of Ch-DNA and Ch-PEG-FA-DNA nanoparticle diameters makes this study unique as very few has elaborated the Gaussian distribution of Ch nanoparticle size [28]. It has been suggested that polydispersity of the Ch used to complex DNA can have an effect on the size of the resulting particle [27].

Two properties are necessary to assure nanoparticle uptake by cells: zeta potential (or surface charge) and size. Previous studies have confirmed that DDA, MW, N : P ratio, and pH do not influence complex size [29]. Nanoparticle size is one of the variables for favorable cellular uptake. Nanoparticles of smaller size have the advantage of entering cells more easily by endocytosis or pinocytosis and crossing nuclear-pore complexes, thereby increasing the transfection rate [15]. It has been proposed that for polycation-DNA gene delivery systems to enter cells, a size requirement below 100 nm is necessary [24].

Our team and others have previously demonstrated that a positive surface charge allows electrostatic interaction between negatively charged cellular membranes and positively charged nanoparticles [16]. Ch-DNA nanoparticle size was indirectly proportional to the charge ratio (N : P) up to 12 mV, while zeta potential was directly proportional to it (N : P). 

A fine balance must be achieved between extracellular DNA protection (better with high MW or HMW) versus efficient intracellular unpacking (better with LMW) to obtain high levels of transfection. HMW Ch can be depolymerized by different methods, such as ultrasound, heat, enzymatic hydrolysis, and chemical hydrolysis. Depolymerization of Ch by nitrous acid is becoming a favored technique since it is economical, rapid and can be controlled to produce Ch of preselected size [29].

In this study, the effect of Ch-DNA and Ch-PEG-FA-DNA nanoparticles on KB cell viability was compared to naked DNA and LF. There was no significant change in cell viability between Ch-treated and untreated KB cells. KB cells incubated with Ch25-PEG-FA-*β*-gal nanoparticles showed 100% cell viability. Also, 90% to 100% cell viability was observed in cells incubated with Ch25-PEG-FA or Ch50-PEG-FA. All Ch nanoparticule combinations showed much lower cytotoxicity compared to LF. 

These results and previous studies confirm that Ch and Ch nanoparticles are nontoxic in a range of toxicity tests, both *in vitro* [24] and in experimental animals [13].

### 4.2. *In Vitro* Transfection Efficiency of Ch Nanoparticles in KB Cells

We compared transfection efficiency to different MW Chs (5, 25 and 50 kDa) and the presence or absence of PEG-FA. Different LMW Chs were used because it was previously demonstrated that DNA can be more easily released from them [30].

The choice of plasmid DNA was based on the evaluation method of gene expression and its corresponding size, either VR1412 (*β*-gal reporter vector for assaying *β*-gal activity) or GFP (emitting green fluorescence when expressed). KB cells were mainly chosen because of FR overexpression [26]. The transfection efficiency of Ch nanoparticles has been studied previously in Cos-1 cells, HeLa cells, Hep-G2 cells, and 293human embryonic kidney cells [13, 24].

Gene expression was significantly higher with Ch25-PEG-FA-DNA nanoparticles. No significant differences were seen when comparing the rate of transfection with LF (positive control) to Ch25-PEG-FA-DNA nanoparticles. 

The transfection efficiency of nonviral vectors may depend on several factors, such as chemical polycation characteristics (DDA, MW, N : P ratio, Ch : DNA ratio, and pH), size and composition of complexes, interaction between cells and complexes, and cell type [15]. We have studied the influence of Ch MW while other factors were kept constant (DDA = 89, pH = 7.4).

It has been demonstrated that gene expression levels are closely related to polymer MW [29]. Binding affinity and complex formation between oppositely charged macromolecules are strongly dependent on the valence of each molecule, with low valence yielding only weak binding. The reduction in Ch valence at LMW has been shown to decrease its binding affinity for DNA and to increase DNA decomplexation and gene expression [29]. 

The percentage of transfected cells significantly depends on the type of complexes used and is sensitive to Ch MW. It was suggested that high MW Ch may form very stable complexes, making it hard to express a given gene sequence since they may not be disassembled once inside the cell. On the other hand, at critical LMW, Ch cannot fully condense DNA [29]. 

The more efficient cell transfection of Ch-DNA complexes compared to naked DNA may be due to a zipper-like association of excess positive charges of the complexes with the negatively charged cell surface. This interaction may result in adsorptive endocytosis and membrane stability [27]. 

PEG-FA nanoparticle complexes (Ch25-PEG-FA-DNA) have been shown to facilitate DNA internalization into cells. High-affinity FR binding is retained when FA is covalently linked via its *γ*-carboxyl group to a foreign molecule. The presence of FR in certain diseases helps to target and deliver DNA to diseased cells while avoiding uptake into normal cells. It is a highly specific and versatile technique that can be applied to a wide variety of drugs and diseases. It is a good strategy to transfect several cancer cell types (such as ovarian and breast cancers) [31]. Activated macrophages are responsible for the progression of autoimmune diseases such as RA, psoriasis, ulcerative colitis, and lupus [18, 19]. Activated macrophages in RA overexpress membrane FR [18, 32] and represent a potential opportunity for FR-targeted gene therapy. The presence of the FA group seems to help internalization in KB cells, probably through their FR.

## 5. Conclusion

We synthesized and then assessed Ch nanoparticles with different characteristics and selected the one best suited for selective transfection efficiency in human KB cells *in vitro. *To the best of our knowledge, this is the first study to confirm that nanoparticules with Ch25 complexed to FA obtain better transfection efficiency in cells overexpressing FR. Our system should have the potential of being employed as specific cell-targeting systems with cells having high FA occurrence.

## References

[1] Porteous DJ, Dorin JR, McLachlan G (1997). Evidence for safety and efficacy of DOTAP cationic liposome mediated CFTR gene transfer to the nasal epithelium of patients with cystic fibrosis. *Gene Therapy*.

[2] Youjin S, Jun Y (2009). The treatment of hemophilia A: from protein replacement to AAV-mediated gene therapy. *Biotechnology Letters*.

[3] Liu P, Santisteban I, Burroughs LM (2009). Immunologic reconstitution during PEG-ADA therapy in an unusual mosaic ADA deficient patient. *Clinical Immunology*.

[4] Braun S (2008). Muscular gene transfer using nonviral vectors. *Current Gene Therapy*.

[5] Ulug P, Vasavda N, Kumar R (2008). Hydroxyurea therapy lowers circulating DNA levels in sickle cell anemia. *American Journal of Hematology*.

[6] Gao X, Kim KS, Liu D (2007). Nonviral gene delivery: what we know and what is next. *The American Association of Pharmaceutical Scientists Journal*.

[7] Mansouri S, Lavigne P, Corsi K, Benderdour M, Beaumont E, Fernandes JC (2004). Chitosan-DNA nanoparticles as non-viral vectors in gene therapy: strategies to improve transfection efficacy. *European Journal of Pharmaceutics and Biopharmaceutics*.

[8] Marsh M, Helenius A (2006). Virus entry: open sesame. *Cell*.

[9] Campbell EM, Hope TJ (2005). Gene therapy progress and prospects: viral trafficking during infection. *Gene Therapy*.

[10] Oligino TJ, Yao Q, Ghivizzani SC, Robbins P (2000). Vector systems for gene transfer to joints. *Clinical Orthopaedics and Related Research*.

[11] Niidome T, Huang L (2002). Gene therapy progress and prospects: nonviral vectors. *Gene Therapy*.

[12] Nishikawa M, Huang L (2001). Nonviral vectors in the new millennium: delivery barriers in gene transfer. *Human Gene Therapy*.

[13] Zhang X, Yu C, XuShi, Zhang C, Tang T, Dai K (2006). Direct chitosan-mediated gene delivery to the rabbit knee joints *in vitro* and *in vivo*. *Biochemical and Biophysical Research Communications*.

[14] Borchard G (2001). Chitosans for gene delivery. *Advanced Drug Delivery Reviews*.

[15] Mao HQ, Roy K, Troung-Le VL (2001). Chitosan-DNA nanoparticles as gene carriers: synthesis, characterization and transfection efficiency. *Journal of Controlled Release*.

[16] Fernandes JC, Tiera MJ, Winnik FM, Kumar CSSR (2006). DNA-chitosan nanoparticles for gene therapy: current knowledge and future trends. *Biological and Pharmaceutical Nanomaterial *.

[17] Turk MJ, Breur GJ, Widmer WR (2002). Folate-targeted imaging of activated macrophages in rats with adjuvant-induced arthritis. *Arthritis and Rheumatism*.

[18] Sabharanjak S, Mayor S (2004). Folate receptor endocytosis and trafficking. *Advanced Drug Delivery Reviews*.

[19] Sudimack J, Lee RJ (2000). Targeted drug delivery via the folate receptor. *Advanced Drug Delivery Reviews*.

[20] Mima S, Miya M, Iwamoto R, Yoshikawa S (1983). Highly deacetylated chitosan and its properties. *Journal of Applied Polymer Science*.

[21] Cho KC, Kim SH, Jeong JH, Park TG (2005). Folate receptor-mediated gene delivery using folate-poly(ethylene glycol)-poly(L-lysine) conjugate. *Macromolecular Bioscience*.

[22] Kiang T, Wen J, Lim HW, Leong KW (2004). The effect of the degree of chitosan deacetylation on the efficiency of gene transfection. *Biomaterials*.

[23] http://www.addgene.org/pgvec1.

[24] Corsi K, Chellat F, Yahia L, Fernandes JC (2003). Mesenchymal stem cells, MG63 and HEK293 transfection using chitosan-DNA nanoparticles. *Biomaterials*.

[25] Fernandes JC, Wang H, Jreyssaty C (2008). Bone-protective effects of nonviral gene therapy with folate-chitosan DNA nanoparticle containing interleukin-1 receptor antagonist gene in rats with adjuvant-induced arthritis. *Molecular Therapy*.

[26] Bozkir A, Saka OM (2004). Chitosan-DNA nanoparticles: effect on DNA integrity, bacterial transformation and transfection efficiency. *Journal of Drug Targeting*.

[27] Jo HG, Min KH, Nam TH, Na SJ, Park JH, Jeong SY (2008). Prolonged antidiabetic effect of zinc-crystallized insulin loaded glycol chitosan nanoparticles in type 1 diabetic rats. *Archives of Pharmacal Research*.

[28] Lavertu M, Méthot S, Tran-Khanh N, Buschmann MD (2006). High efficiency gene transfer using chitosan/DNA nanoparticles with specific combinations of molecular weight and degree of deacetylation. *Biomaterials*.

[29] Turan K, Nagata K (2006). Chitosan-DNA nanoparticles: the effect of cell type and hydrolysis of chitosan on *in vitro* DNA transfection. *Pharmaceutical Development and Technology*.

[30] Reddy JA, Haneline LS, Srour EF, Antony AC, Clapp DW, Low PS (1999). Expression and functional characterization of the *β*-isoform of the folate receptor on CD34^+^ cells. *Blood*.

[31] Lee D, Lockey R, Mohapatra S (2006). Folate receptor-mediated cancer cell specific gene delivery using folic acid-conjugated oligochitosans. *Journal of Nanoscience and Nanotechnology*.

[32] Nakashima-Matsushita N, Homma T, Yu S (1999). Selective expression of folate receptor *β* and its possible role in methotrexate transport in synovial macrophages from patients with rheumatoid arthritis. *Arthritis and Rheumatism*.

